# Supplementary material for: Network Analysis of Enhancer–Promoter Interactions Highlights Cell-Type-Specific Mechanisms of Transcriptional Regulation Variation
Source: Int J Mol Sci. 2024 Sep 11;25(18):9840. doi: 10.3390/ijms25189840 (PMC11432627; doi:10.3390/ijms25189840)
Supplement: Supplementary file 1 [file ijms-25-09840-s001.zip › Supplemental_Figure_Legends.pdf]

Supplemental Figure S1: EPI statistics for cell-types: Box plot showing similar distribution of ABC scores for the EPIs among the different cell types.

Supplemental Figure S2: Shared regulatory elements across cell types: (A,B,C) Upset Plots show the number of elements per cell type (horizontal barplot), the cell types in the interaction (filled in dots on bottom), and the number of elements found in the cell types denoted (vertical barplot). Rectangles separate the interactions based on the number of cell types included in the intersection set. Plots are done for EPIs (A), Enhancers only (B), and Promoters only (C). These plots highlight that EPIs and enhancers are largely cell type specific and the majority of overlap is within differentiation stage, while promoters are less cell type specific and are commonly found across all types of cell type intersections.

Supplemental Figure S3: Shared Overlap of regulatory elements across subset EPI networks. (A) Barcharts display the percentage of enhancer and promoter regions that are only found within that cell type subset. (B) Table containing jaccard index values of shared enhancers (bottom left) and promoters (upper right) between any two cell types. Color gradient is separately calculated for each regulatory element, with darker cells representing higher levels of shared elements between the two cell types.

Supplemental Figure S4: Transcription Factor cell type gene expression: (A) Heatmap displaying the RNA transcript per million (TPM) values per cell type (rows) for each transcription factor (column). Red cells denote higher TPM for that transcription factor in that cell type compared to the other cell types. All columns have been organized by hierarchical clustering to identify patterns of TF TPM values across cell types. TFs are then grouped into the cell type that contains the highest TPM value for that TF. (B) Gene Ontology Enrichment Analysis of the six grouped TFs. Note that the ESC cell type fails to have any significant enrichments below the q-value < 0.05 threshold and as such is not included in the figure. Additionally the NSC cell type only finds 6 processes passing this threshold. (C) Gene Ontology Enrichment Analysis of the TFs after combining TFs within differentiation stage.

Supplemental Figure S5: Binary matrix of transcription factor (TF) presence. Binary heatmap displaying enhancers as rows and TFs per column. Red cells denote presence and blue cells denote absence. Rows are grouped by cell type and columns are clustered via hierarchical clustering. We find there are no enhancers that are uniquely found in a certain cell type or any significant patterns of binding that separate the cell type based on the TF raw binding presence alone.

Supplemental Figure S6: Empirical overlap of ASD case (A,C,E,G,I) and control (B,D,F,H,J) variants between cell type EPI enhancers and randomly selected EPI enhancers. Histogram plots for empirical analysis of enhancer regulatory regions for the 5 non-representative cell types containing ASD patient identified de novo variants. The X-axis of the histograms represents the number regions containing variants. The Y-axis of the histograms represents the frequency of the 1000 randomly selected iteration observed values. The red line represents the observed number of enhancer regions containing case or control variants in that cell type's EPI network. Each row of the figure is 1 cell type and the left column are the case variant analysis histograms and the right column are the control variants analysis histograms

Supplemental Figure S7: Empirical overlap of ASD case (A,C,E,G,I) and control (B,D,F,H,J) variants between cell type EPI promoters and randomly selected EPI promoters. Histogram plots for empirical analysis of promoter regulatory regions for the 5 non-representative cell types containing ASD patient identified de novo variants. The X-axis of the histograms represents the number regions containing

variants. The Y-axis of the histograms represents the frequency of the 1000 randomly selected iteration observed values. The red line represents the observed number of promoter regions containing case or control variants in that cell type's EPI network. Each row of the figure is 1 cell type and the left column are the case variant analysis histograms and the right column are the control variants analysis histograms.

Supplemental Figure S8: Empirical overlap of ASD associated genes (A,C,E,G,I) and NDD associated genes (B,D,F,H,J) variants between cell type EPI target genes and randomly selected EPI target genes. Histogram plots for empirical analysis of target gene regions for the 5 non-representative cell types containing target genes previously associated with ASD or NDD. The X-axis of the histograms represents the number target genes that have been previously associated with ASD or NDD. The Y-axis of the histograms represents the frequency of the 1000 randomly selected iteration observed values. The red line represents the observed number of target genes previously associated with ASD or NDD in that cell type's EPI network. Each row of the figure is 1 cell type and the left column are the associated ASD gene analysis histograms and the right column are the associated NDD gene analysis histograms

Supplemental Figure S9: Empirical overlap of SCZ variants in EPI enhancer regions (A,C,E,G,I) and promoter regions (B,D,F,H,J) variants between cell type EPIs and randomly selected EPIs. Histogram plots for empirical analysis of enhancer or promoter regulatory regions for the 5 non-representative cell types containing SCZ GWAS variants. The X-axis of the histograms represents the number regions containing variants. The Y-axis of the histograms represents the frequency of the 1000 randomly selected iteration observed values. The red line represents the observed number of enhancer or promoter regions containing SCZ variants in that cell type's EPI network. Each row of the figure is 1 cell type and the left column are the enhancer region analysis histograms and the right column are the promoter region analysis histograms
