# Supplementary figures and images for: Network Analysis of Enhancer–Promoter Interactions Highlights Cell-Type-Specific Mechanisms of Transcriptional Regulation Variation
Source: Int J Mol Sci. 2024 Sep 11;25(18):9840. doi: 10.3390/ijms25189840 (PMC11432627; doi:10.3390/ijms25189840)

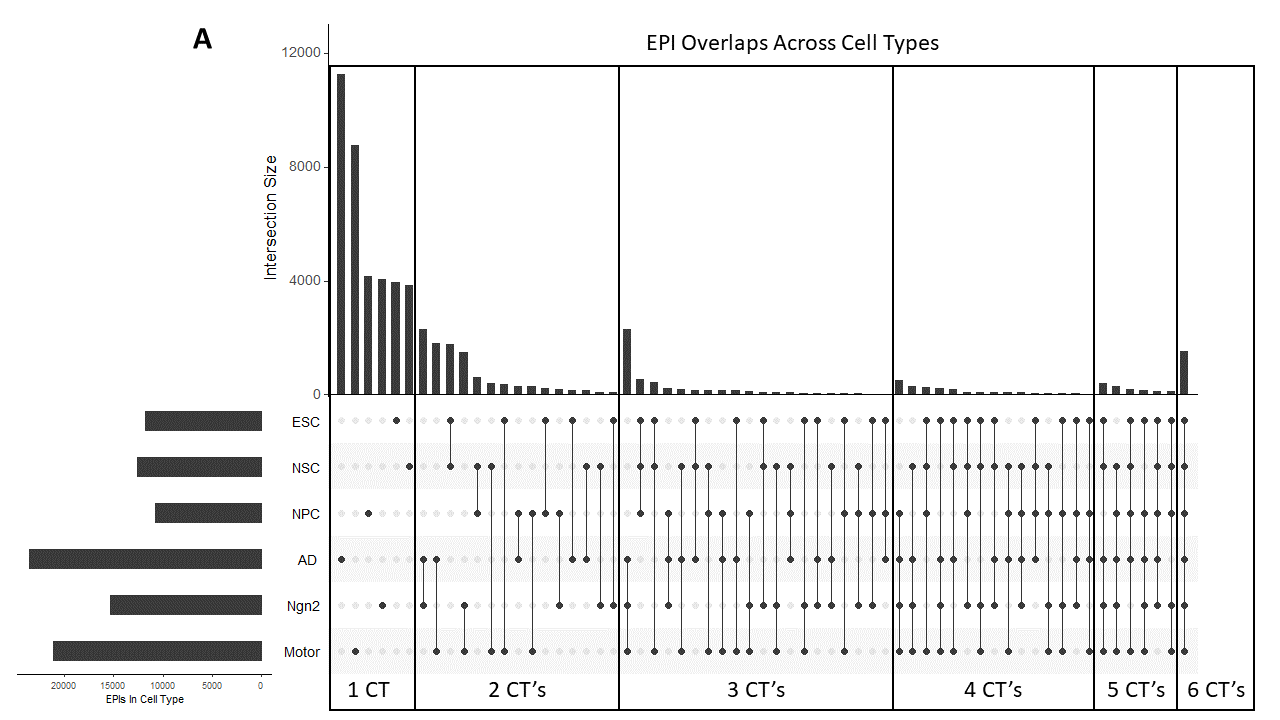

Supplement: Supplementary file 1 [file ijms-25-09840-s001.zip › S2A_Fig.tif]

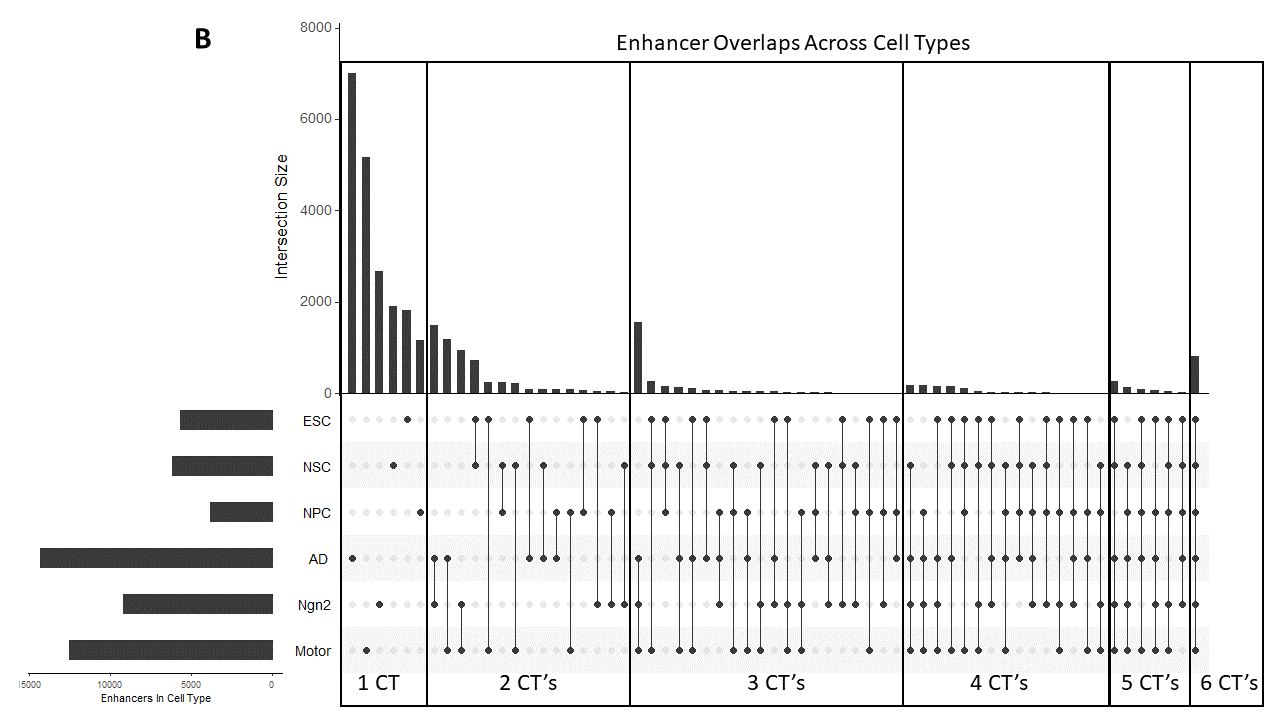

Supplement: Supplementary file 1 [file ijms-25-09840-s001.zip › S2B_Fig.tif]

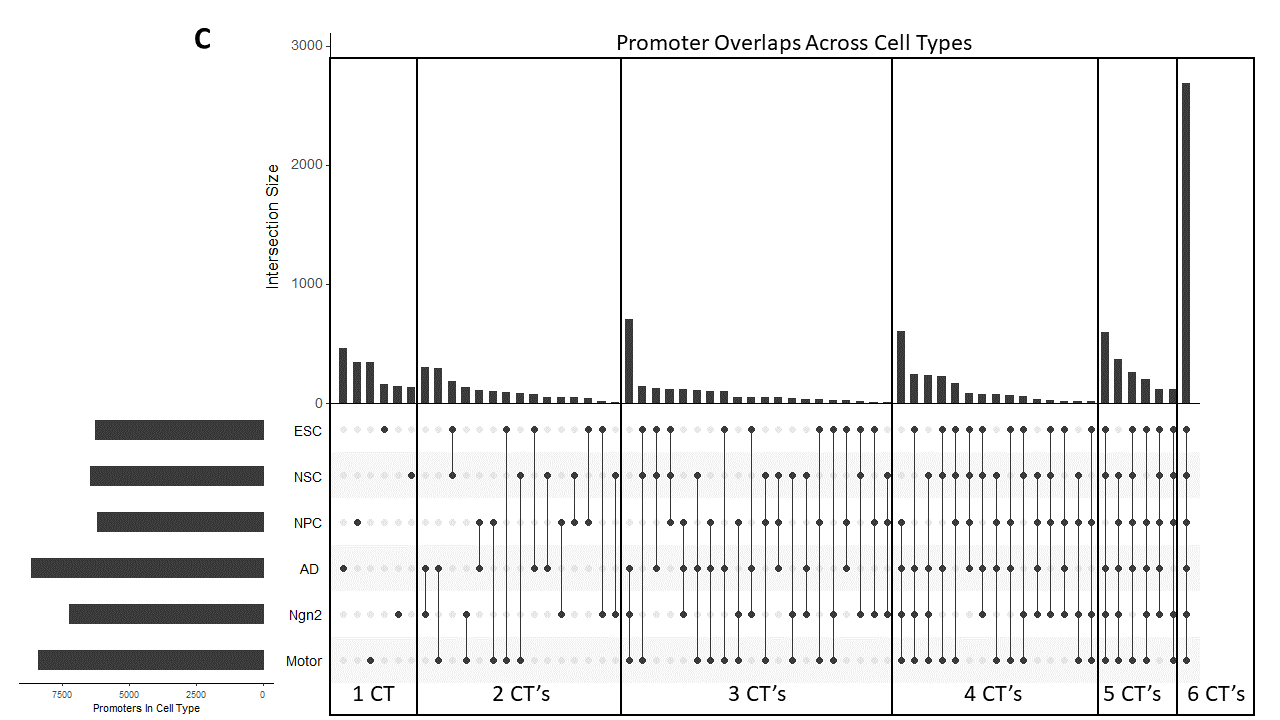

Supplement: Supplementary file 1 [file ijms-25-09840-s001.zip › S2C_Fig.tif]

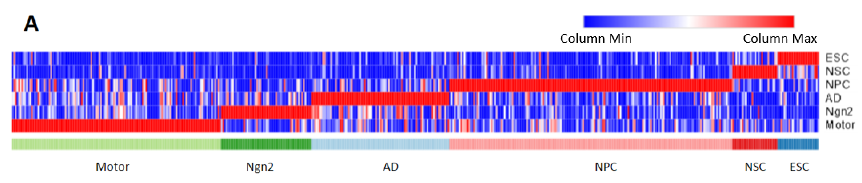

Supplement: Supplementary file 1 [file ijms-25-09840-s001.zip › S4A_Fig.tif]

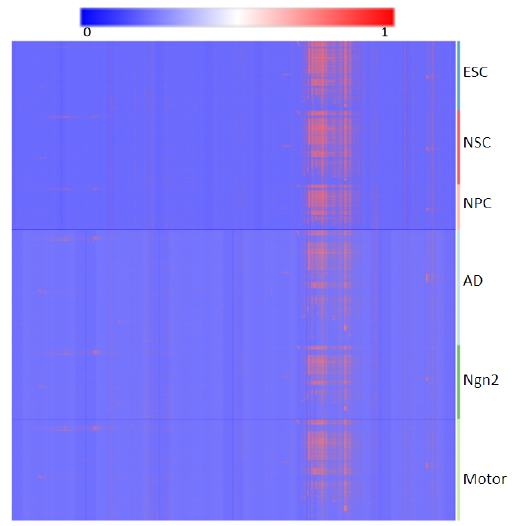

Supplement: Supplementary file 1 [file ijms-25-09840-s001.zip › S5_Fig.tif]
